# Introducing methadone maintenance therapy into Ukrainian prisons: a qualitative study of criminal subculture, Russia’s full-scale invasion, and contested methadone objects

**DOI:** 10.3389/fpsyt.2023.1227216

**Published:** 2023-11-30

**Authors:** Matthew Ponticiello, Lyu Azbel, Mary M. Tate, Daniel J. Bromberg, Iryna Pykalo, Tetiana Kiriazova, Natalya Saichuk, Frederick L. Altice

**Affiliations:** ^1^Yale School of Medicine, Yale University, New Haven, CT, United States; ^2^Department of Epidemiology of Microbial Diseases, Yale University, New Haven, CT, United States; ^3^Department of Social and Behavioral Sciences, Yale University, New Haven, CT, United States; ^4^European Institute on Public Health Policy, Kyiv, Ukraine; ^5^Ukrainian Institute on Public Health Policy, Kyiv, Ukraine

**Keywords:** Ukraine, HIV prevention, qualitative, methadone, prisons, Russia, war, conflict

## Abstract

**Background:**

After pilot testing, methadone was newly being introduced into Ukrainian prisons in 2021 as part of a national scale-up strategy to treat opioid use disorder and prevent transmission of HIV and HCV infections. Opioid agonist therapy (OAT) scale-up in Eastern Europe and Central Asia prisons has been hampered by varying levels of influence of criminal subculture, an extralegal *informal governance* by a social hierarchy that operates in parallel to formal prison authorities. This study examined the socio-environmental factors influencing the uptake of methadone treatment in Ukrainian prisons, including changes that evolved during Russia’s full-scale invasion of Ukraine and the displacement of people deprived of liberty (PDL) from conflict to non-conflict regions.

**Methods:**

In-depth qualitative interviews (*N* = 37) were conducted from January 2021 to October 2022 in the only two Ukrainian prisons where methadone was being introduced with PDL (*N* = 18). These two prisons continued to provide methadone after the full-scale invasion. Former PDL (*N* = 4) were also interviewed and prison staff (*N* = 15). Interviews were audio-recorded, transcribed, and translated into English. Four authors independently reviewed, coded, and applied a phenomenological framework for data analysis, delineating themes related to criminal subculture, drug use, methadone uptake, and evolving changes during the Russian invasion.

**Findings:**

Criminal subculture perceptions varied, with some seeing it as strongly discouraging drug use among certain groups, while others described it as a residual and weak influence from a more distant past. The influence of the subculture on methadone treatment uptake, however, was less clear. PDL and prison staff struggled to identify and articulate differences between illicit street-bought methadone, used recreationally, and medically prescribed methadone. Thus, the meaning of “methadone” varies in interpretation as it is being introduced, making it potentially conflicting for patients to opt into this evidence-based treatment. As Russia invaded Ukraine in 2022, PDL from conflict zones were transferred to non-conflict regions where methadone was being introduced. The prison environment became more enabling for PDL to start methadone as they were segregated and not subject to the existing criminal subculture’s rules and lacked the social ties necessary to procure drugs illegally.

**Conclusion:**

It appears that the criminal subculture is variable and evolving in Ukrainian prisons and appears to be impacted differently by the invasion of Russia. As methadone scale-up in prisons expands, it will be important to distinguish the meaning of methadone perpetuated negatively by the prison subculture versus that in which it is intended as a medical treatment by the formal prison authorities. The current invasion of Ukraine by Russia provides a potential disruption to alter this course.

## Introduction

The criminalization of drug use concentrates people with opioid use disorder (OUD) and blood-borne infections like HIV (PWH) and hepatitis C virus (HCV) in prisons (1, 2). Moreover, as people who inject drugs (PWID) enter prison, within-prison drug injection often continues (3–6), including in Ukraine (7), resulting in outbreaks of HIV and HCV within prisons (8). The syndemic nature of HIV, HCV, drug injection, and incarceration (9) is especially salient in Eastern Europe and Central Asia (EECA), where, unlike elsewhere globally, HIV mortality and incidence continue to increase, fueled by the sharing of injection equipment and high rates of incarceration (10, 12).

Maintenance with opioid agonist therapy (OAT), using methadone (MMT) or buprenorphine, is the most effective treatment for opioid use disorder (13) among PWID, substantially reducing mortality and transmission of blood-borne infections (13, 14). Introducing and scaling up OAT within the prison, when combined with an effective linkage program to community treatment, contributes to country-wide scale-up of OAT as most PDL return to their communities (15, 16). Despite the increasing availability of OAT programs, they are substantially more limited within prisons and primarily use MMT as it is the least expensive (2). Until 2020, prison-based MMT in EECA, out of all countries in the EECA region, was provided only in Moldova, the Kyrgyz Republic, and Armenia (8). In 2020, MMT was introduced as a pilot study in Ukraine and Tajikistan; buprenorphine was introduced in Georgia. Suboptimal implementation of OAT within prisons, alongside the disabling HIV risk environment, the legal framework, criminal subculture, and the perception that OAT is ineffective, has undermined OAT scale-up within prisons (17). Moreover, misinformation and negative attitudes toward OAT by prison administration personnel (18) and people deprived of liberty (PDL) (15, 19–21) continue to impede scale-up.

Ukraine has the second highest number of PWID in EECA and a high HIV prevalence (19–26%) among PWID (22), making it crucial to scale-up OAT (14), including in prisons. As Ukraine introduced its first pilot methadone program to 38 PDL in a single prison starting in 2020, there was little known about the within-prison context. Within EECA, though described elsewhere (e.g., gangs in North America) (8, 23, 24), criminal subcultures exist with varying degrees of governance within the prison. These criminal subcultures include status hierarchies, and their systems of governance have the potential to greatly influence the behaviors of PDL within the prison system. Additionally, the criminal subculture provides necessary resources to PDL like personal goods that are not provided by the formal prison authorities (25). Though the social order that evolves among PDL is not entirely unique to post-Soviet nations, its presence in Ukraine (26, 27), Moldova (21), and Kyrgyzstan (28) is a particular phenomenon (29). In Moldova and Kyrgyzstan, where methadone treatment provided in prison has been present for nearly two decades, criminal subculture is described as especially powerful, and it has greatly inhibited OAT uptake (19, 28). Little data about these hierarchies in Ukrainian prisons exist and as MMT is being expanded to other prisons there, it will be crucial to understand how these informal hierarchies work and how, if at all, they may influence MMT uptake in Ukrainian prisons. Moreover, as MMT is scaled up in Ukraine, internal prison dynamics may change as a result of Russia’s full-scale invasion of Ukraine, further influencing the OAT scale-up (30).

As there are data before MMT was introduced that a prison subculture existed within some Ukrainian prisons (26), this qualitative study was initially conducted as part of an implementation trial to newly introduce and scale up methadone within two Ukrainian prisons. The two prisons selected for this study are the only two where MMT was introduced before the invasion and, due to their location away from the conflict, continued to provide MMT after the war. The key question among multiple stakeholders (PDL, recently released PDL, NGO staff, and prison administrators) was to what extent might the criminal subculture influence methadone scale-up and whether the way that methadone is perceived by PDL evolves in meaning as an effective treatment for opioid use disorder or devolve as an intoxicant similar to non-prescribed drugs. As the time period involved the invasion of Ukraine by Russia, it also provided an opportunity to examine how the war influenced how receptive the within-prison context was toward methadone as individuals came under more psychological distress from the war.

## Methods

### Study setting and design

Until 2016, Ukraine had one of the highest incarceration rates worldwide. The prison census decreased from 160,000 to 49,000 by 2021 due to legislative changes and the introduction of a new probation system starting in 2016. Pre-decarceration data from 2012 showed that among PDL in Ukraine, HIV prevalence was 19.4% and 48.7% are PWID, with over a third meeting the criteria for OUD (30). No new rigorous nationally representative biobehavioral surveys have been conducted since. The 124 prisons in Ukraine are divided into those with first and recidivist incarcerations, as well as varying security levels (low, medium, and high). The pilot program to introduce methadone in PDL included 38 participants in Bucha; this prison has since closed. Plans for introducing and scaling up methadone were then planned for nine dedicated prisons and pre-trial detention centers, including the two we studied.

Qualitative, in-depth interviews were conducted with a number of key stakeholders directly or indirectly involved in the prison-based methadone program (31). The two prisons assessed were medium security and located in Central Ukraine, with one (*N* = 540) being a prison for recidivists while the other (*N* = 415) being for first-time offenders.

The 2022 invasion of Ukraine by Russia resulted in major shifts in the population. PDL from the frontline regions in the East were transported to the West, where they were housed within the western prison territories but segregated from the other original resident PDL at those prisons. This segregation was purposeful from a security perspective to reduce the potential for tension between existing residents and large numbers of newly arrived PDL.

### Sampling and recruitment

Using purposive snowball sampling (32), we identified and recruited participants from five key groups in two Ukrainian prisons: (1) prison administrators; (2) clinicians associated with the methadone treatment program; PDL either (3) receiving methadone or (4) not receiving methadone; and (5) NGO staff working outside the prison. Purposive sampling was done to identify participants with characteristics of interest (such as status in the hierarchy and experience with OAT) and to recruit approximately the same number of PDL receiving and not receiving methadone. Eligibility includes those aged ≥18 years from one of the groups and provided informed consent to be audio-recorded. With the assistance of prison administrators, prison staff—clinicians associated with the methadone treatment program and administrators—were identified and recruited from the two prisons in Ukraine (*n* = 15). Prison staff were included in this study as they consistently interact daily with PDL and could provide outsider perspectives on the dynamics between PDL. In addition, prison staff can markedly influence the shaping of the prison environment, which consequently influences the implementation and uptake of methadone among PDL. PDL enrolled in the methadone program (*n* = 10) and PDL not enrolled in the methadone program (*n* = 8) were also identified and recruited in the same two prisons. The final sample interview was conducted until saturation of themes was observed. Both PDL enrolled and not enrolled in the methadone program were included in this study as we sought diverse perspectives and wanted to identify if there were common themes among PDL who chose to engage in the methadone program versus those who did not. Participants were recruited by being called to the doctor’s office by medical staff, where they were told about a voluntary research study about drug use, addiction treatment, and health. A research assistant then explained the study to them and performed consent procedures. Participants were recruited to understand a range of experiences and perspectives, and interviews continued until thematic saturation was reached (33–37). Former PDL (*n* = 4) were also recruited as a part of this study. Former PDL were included in this study to elucidate if any unique changes in perceptions of prison-based methadone occurred post-release and reduce any potential bias of these perceptions that PDL might not want to disclose while still within the prison setting. There were two interviewers who spoke Russian (DJB and MMT) and one who spoke Ukrainian and Russian (LA). Participants chose the language they preferred to speak in. In-person interviews were conducted in the medical administrative facility. This potentially deterred some PDL of higher status from participating as it may have been seen as collaboration with the formal administration. During COVID and after the start of the full-scale invasion, interviews were conducted via video link. In video interviews, participants wore noise-canceling headphones in private rooms within the prison’s medical facility.

### Data generation

Between January 2021 and October 2022, participants were initially invited to partake in a semi-structured interview guide that was expanded over time to in-depth interviews that explored how criminal subculture interfaces with drug use in the Ukrainian prison setting and how participants understood and experienced the prison-based methadone program to ensure consistency across interviews (Appendix 1). The open-ended nature of the questions allowed interviewers to inquire about the impacts of the Russian invasion when appropriate, as well as any other emerging themes. Before fieldwork, semi-structured guides were pilot-tested with one member of each stakeholder group. Data generation during the pilot test was excluded from the study dataset. The interviews were conducted via Zoom by three experienced researchers fluent in Ukrainian, Russian, and English in a private room of the prison medical facility.

### Data analysis

Thirty-six interview transcripts were analyzed as part of a single dataset, as one interview was not recorded. Professional Ukrainian translators fluent in Ukrainian, Russian, and English transcribed the audio recordings verbatim in the appropriate language (Ukrainian or Russian) and then into English for analysis. Author LA reviewed all Ukrainian and Russian to English transcripts for quality assurance. Four authors (LA, MP, MMT, and DJB) independently reviewed the transcripts and created a coding scheme relevant to the criminal subculture, methadone treatment, and the Russian invasion. A phenomenological framework was used for data analysis as we sought to understand participant perspectives on an experiential phenomenon of interest; in this case, the role of a criminal subculture within Ukrainian prisons, within the context of participants’ lives. We follow this framework to ask what is meant by the phenomenon of a criminal subculture and how it operates in the lived experience of people in Ukraine to shape their relationship to methadone treatment. Subjective understandings of the criminal subculture shape decision-making regarding the utilization of program methadone, hence the utility of this framework (38–41).

An open-coding approach was used to develop codes that would be subsequently refined through constant comparison (42). All authors discussed and agreed on the final coding scheme. Final codes were grouped into themes and analyzed using a content analysis approach (43). Finally, representative quotations were selected to illustrate the study findings and to draw out themes regarding the influence of criminal subculture on methadone treatment uptake and the effects of the Russian invasion on prison-based methadone treatment programs.

### Ethics approvals

This study received ethics approvals from the Ukrainian Institute on Public Health Policy IRB (Protocol no. 2016-031-13) and Yale University IRB (Protocol no. 1407014374). Heads of prison administration provided consent for recruiting clinicians, workers, and PDL at their prisons. All participants provided written consent to participate in the interview. A copy of the signed consent form was given to the participant for their records. All participants received de-identified study numbers to maximize confidentiality. Each participant received a hygiene kit as compensation for their time.

## Results

### Characteristics of study participants

All 37 participants interviewed in two Ukrainian prisons were included in this study. Each person invited to participate agreed and completed an interview. A summary of participant characteristics can be found in Table 1. All former and current PDL were men (*N* = 22, 100%), and three staff (20%) were women. Among the 37 interviews, 48.6% were conducted with current PDL. Ages ranged from 25 to 43 years old, with a median age of 34 years. Most PDL had been incarcerated on average for 3.3 years.

**Table 1:** Participant characteristics.

Characteristics	People in prison (*n* = 18)	Former people in prison (*n* = 4)	Prison staff (*n* = 15)
Male, no. (%)	18 (100)	4 (100)	12 (80)
Age (years), median (IQR)	34 (31–41)	32.5 (28.5–38)	n/a
Ever enrolled in a methadone program, no. (%)	10 (55.5)	1 (25)	n/a

### Summary of results

This qualitative study aims to elucidate the role of a criminal subculture in Ukrainian prisons where methadone treatment was newly introduced for PDL. First, we describe how informal prison structures relate to drug use. Next, we examine conceptualizations of “street methadone” versus “program methadone.” Among PDL, we observed that the two are often viewed as distinct. Street methadone refers to the methadone people obtain illegally and use outside of a treatment setting in the community (typically injected), while program methadone refers to the liquid methadone offered through methadone treatment programs. Third, we note the changing role of the criminal subculture and shifts toward more Western models of prison governance where informal hierarchies wield less power. Finally, we report on the effects of the Russian invasion on methadone treatment in two Ukrainian prisons.

#### Understanding how informal prison structures relate to drug use

**Figure 1:**
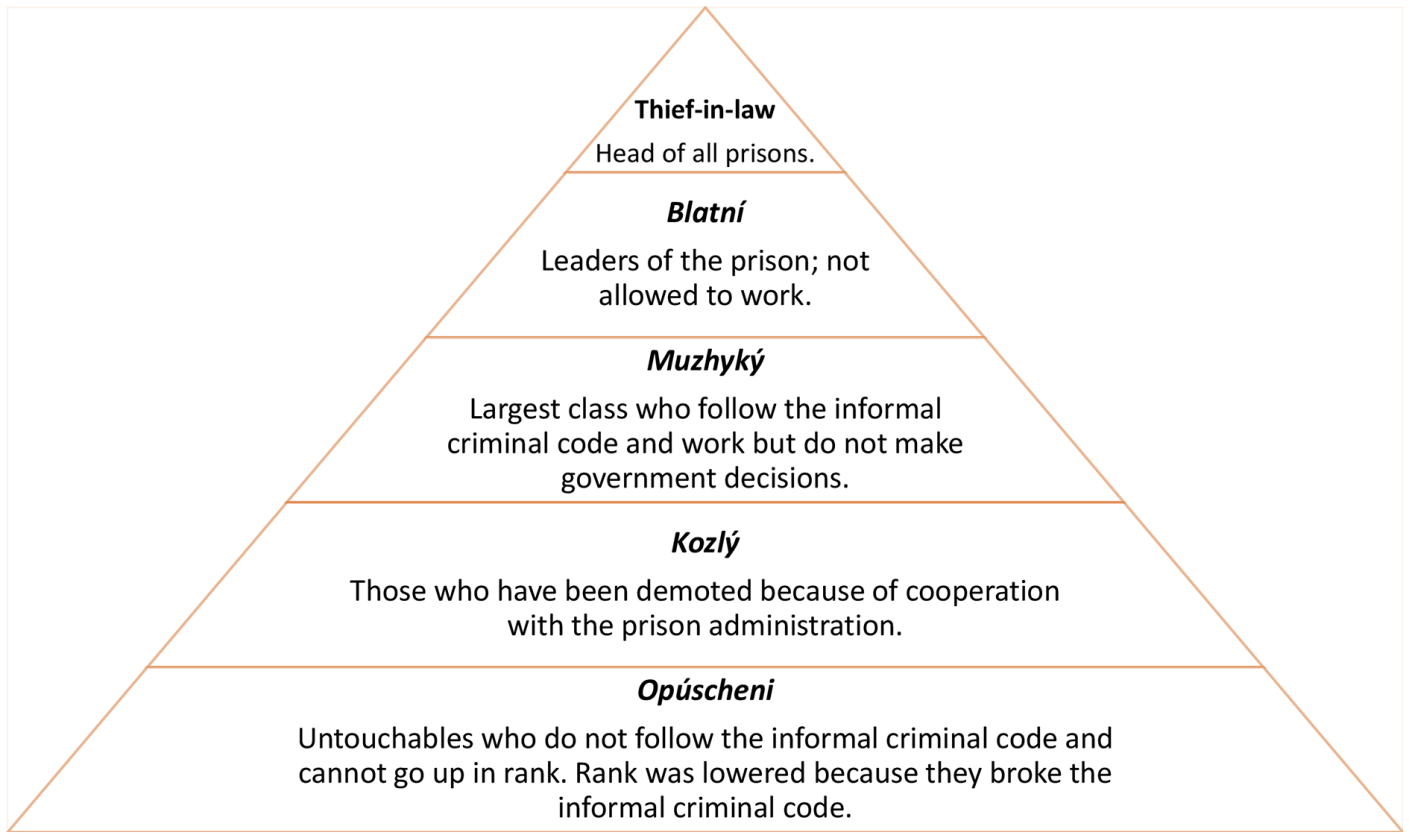
Informal criminal code hierarchy in Ukrainian prisons.

There was variability in the perception of the strength of the informal prison governance. While several participants acknowledged the existence of informal prison structures among PDL, others rejected the notion that criminal subculture heavily influenced PDL’s drug use. PDL described these structures as hierarchal, with many reporting being from either the low or high caste. The hierarchy is as follows: Thief in Law, *blatní* (leaders of the prisons), *muzhyký* (largest class, laborers), *kozlý* (those who have been demoted for cooperating with the prison administration), and *opúscheni* (bottom of the hierarchy and no longer follow the rules, “untouchables”) (Figure 1). In some cases, participants suggested that the status in the hierarchy they occupied could influence their ability to use drugs. Those higher up in the hierarchy typically have more restrictions on their drug use. In contrast, the lower-status PDL have more freedom as it pertains to drug use. One person from a lower caste remarked that they were free to use drugs if they chose to do so.

“Well, my caste is the lowest in the hierarchy, here. I mean, I communicate well with the highest hierarchies. Very good, because I set myself up like this. I mean, in my case, it doesn’t matter. I mean, me, yeah, if I’m gonna do drugs of any kind, no one’s gonna tell me anything. Here, but maybe there are others to whom they may say something…” —Vyktor (PDL not enrolled in OAT, 29 years old, Prison A).

In contrast, prison staff reported the opposite. Prison staff thought that PDL who occupied lower castes in the hierarchy were forbidden to take drugs and that those higher in the hierarchy could use them more freely.

“Talking about the hierarchy, it is blatní and their environment that basically have access to injectable drugs… It is generally not allowed for the opúscheni and for an ordinary hard-working prisoner according to the hierarchy of the colony. They can get punished for it by their own folk…” —Leonid (clinical staff, Prison A).

Participants would refer explicitly to the “thieves’ law” or “thieves’ code.” This code governs criminal subculture and sets limitations on all aspects of life, including drug use. In contrast to low-status PDL, some participants explained that PDL belonging to higher levels in the hierarchy were not allowed to use drugs.

“The problem is that, according to their, let’s say, thieves’ laws, the blatní is the one who looks after the unit, after, well, and so, there, they do not have the right to use any narcotic substances. They decide, as they say, people’s fates, they have to be of sound mind. Thus, drugs are forbidden for them on the whole. It is forbidden to use any drugs according to their laws…” —Mila (prison administrator, Prison B).

Drug use appeared prohibited among the higher-ups because they had more influence and responsibility over others’ “fates,” alluding to the governing responsibility of the higher-ups and the potential for drug use to disrupt their governance. It also was part of a moral code that was expected of those in the hierarchy.

“Like, not everyone should use [drugs]. Understand that, we have a hierarchy in this system. You know? From the little person to the big one. If he’s a big man, he decides people’s fates – he can’t use. He can’t use even a small amount, he must have a 100% consciousness. So, he can’t use and he won’t use” —Anton (PDL enrolled in OAT, 34 years old, Prison B).

Other participants noted that their drug use could threaten their position in the caste system. This phenomenon was described among those in higher castes.

“R: In general. According to the notions, the blatní cannot be using. When I started using it, I was no longer there…They didn't want to push me away, but I said I was using myself…

I: When you started using again, once more, were you able to stay in that caste?

R: I stayed, but unofficially, everyone knew I was there, but I didn't shout out about it, figuratively speaking. But according to our law – it is impossible. You have no right” —Vamava (Former PDL not enrolled in OAT, 36 years old, Prison B).

Some remarked that methadone treatment, specifically, was not an exception to this rule. Methadone treatment was likened to using drugs and was therefore prohibited among members of the informal prison hierarchy. The criminal subculture, in some instances, was understood to dissuade or actively stop PDL from entering methadone treatment.

“Well, each society has its understanding of life and has its principles and concepts that they would not want to be crossed… and they don’t want to allow, for example, those from the [methadone] program to be among them. They will achieve this by some other methods, and so on. There, bypassing the police, not listen to the administration” —Aleksander (former PDL enrolled in OAT, 28 years old, Prison A).

“Well, they [the hierarchy] are basically against it. They do not welcome drug addicts in their circles, well … There are people who use, but they kind of do not welcome it all. Opium is possibly okay, but methadone… not, in their own circles” —Peter (former PDL not enrolled in OAT, 40 years old, Prison B).

While some participants did note the role of the thieves’ code and the criminal subculture in delimitating rules surrounding drug use, others rejected this notion. Some explained that the rules of the hierarchy have begun to take a “back seat.” Consequently, seeking methadone treatment among some PDL was not understood to be at odds with the informal hierarchy.

“There's no such thing [as the hierarchy] now, now if you cooperate with the administration, you cooperate. If you want to be treated [with methadone], you can be treated, no convict will say anything to you, because now all these their concepts, their beliefs, have started to take a bit of a back seat… If the convict wants to be treated, let him be treated” —Mila (prison administrator, Prison B).

The study prisons were located in western or central Ukraine, and the Russian invasion began in the East. As a result, PDL in the East were transferred to central or western prisons as Russia continued to move in on eastern Ukrainian territory. The prison in Vinnytsia was cordoned off to allow for PDL from the East to be housed but segregated from the locals. New PDL who were recently transferred to western prisons from the East because of the war also noted the limited influence of the criminal subculture.

“We lived like people. And here it's not clear at all. The blatní here, they have some kind of rules, but no one respects them” —Taras (newly transferred PDL not enrolled in OAT, 32 years old, Prison A).

#### Conceptualizations of street methadone versus program methadone

Interviews produced blurred boundaries between “street” (illegal) and “program” (prescribed) methadone. Program methadone refers to the liquid methadone administered by prison clinical staff that is given to PDL participating in the prison-based methadone treatment program. Many struggled to identify and articulate the differences between street and program methadone, although they acknowledged the two were different. Methadone was conceptualized by some as a nameless, ambiguous substance.

"Well, we call it methadone. But it's not that kind of methadone… Well, crystal, it's methadone… they just call it crystal, and actually they call it methadone too, but it turns out that methadone is a little bit another concept, as I already understood after the lecture… That's the problem, it doesn't have any particular name, this drug" —Mila (prison administrator, Prison B).

Prison staff reported that some PDL would consider the program methadone a “legal drug.” To delineate the difference between street and program methadone, some prison staff would try to differentiate the two by providing information on the medicinal effects of the program methadone.

“Some inmates ask, ‘are they going to give us legal drugs?’ I explain to them that the methadone they're going to give you, it's not that kind of drug, it's just going to block those receptors that cause you to have that craving for drugs” —Mila (prison administrator, Prison B).

During interviews, participants often explained that they were not on “methadone” but, rather, “street methadone.” The greatest differences between the two are how they were taken and their concentration—street methadone was injected, bought illegally, and more concentrated. In contrast, program methadone was imbibed under clinical supervision.

R: No, I wasn’t on any methadone. I was on, like you said, street methadone, yeah.

I: What do you call it if it’s not street?

R: Methadone.

I: Just methadone?

R: Yes.

I: And your opinion or that of others, what is the difference between program methadone and street methadone?

R: Well, for example, program methadone, we drink it, right? And street methadone, we shoot. That’s the difference. And the difference is great —Vyktor (PDL not enrolled in MMT, 29 years old, Prison A).

The same participant continued to say that he could differentiate the different highs between the two forms of methadone. This was understood to be, in part, due to how the drugs are taken.

R: Yes. From the program methadone, is not high. It acts in about twenty minutes, half an hour. And the street – it immediately acts, because through the vein injected, here. Although through the vein when injected with street methadone it evaporates faster, that is, it passes faster, the effect. And street methadone keeps longer.

I: Does the street methadone? keep longer?

R: Yes. Because it’s absorbed into the stomach. So it keeps longer. I mean, it’s different. And in the other thing, I don’t see the difference —Vyktor (PDL not enrolled in OAT, 29 years old, Prison A).

Other participants felt that street methadone was more addictive and worse for the body. Additionally, participants noted the social stressors that accompanied street methadone, such as financial concerns and potential jail time, and described methadone as free of these consequences.

“Firstly, more addiction, more pulling [from street methadone], it’s worse for the body, much worse than the program [methadone]. And firstly, this is a constant problem, looking for money, not working, there is no way to support yourself. When you're on drugs, there's nothing you can do. It is easier for people on the program, they can work already, they can just get their dose, and they don’t have to look for money or problems. It doesn't lead to jail” —Aleksander (former PDL enrolled in OAT, 28 years old, Prison A).

Furthermore, as “street methadone” availability preceded the introduction of program methadone, the participants began to conflate them, believing in some instances that they were the same. In contrast, others believed them to be different. This blurring of meaning has the potential to disrupt the way that methadone provided within a treatment program could be as negative as illegal “street methadone” and undermine uptake. One participant considered the physical sensations the same but noted that taking methadone through the treatment program transformed the psychological and mental experience.

“Although they say that methadone, well, the OAT program, the drug is the same crystal methadone… on my own, I can say that’s far from true…The feelings are the same. Although physically the same, but psychologically and mentally [different] when you sit in the system” — Ehor (PDL enrolled in OAT, 43 years old, Prison B).

Prison staff commented on how the program methadone, administered by the prison staff, may be uniquely conceptualized among potential users. While drug use in prison occurs, it was understood to be prohibited by the PDL’s subculture, and it was unclear whether methadone fit into this framework as an “illegal” drug or a medication prescription. The legality of using methadone among PDL was further complicated by the fact it was distributed by the prison administration. This, in turn, may have been seen as cooperation with the administration, which would threaten one’s position in the prison hierarchy.

“The inmate’s main point of concern is, especially if he is, so to speak, on the other side of the barricade, that he will become vulnerable and weak after joining the treatment program… And it corresponds with the culture. It’s contraband, it’s under the proper inmate culture, let’s say so, this is normal. But methadone is different, it’s a legal narcotic, it comes from the prison administration. It’s distributed by the administration. If it was done by an outsourced doctor, well, perhaps it would somehow be different. It could be something totally different, it could be cool, you have a doctor from the outside making daily visits. A doctor from the outside comes to you and gives you, like, narcotic drugs. But, it’s like, it’s a whole different ball game…” —Stepanida (NGO staff).

The program methadone is conceived as being different in terms of how you ingest it, its intoxicant properties, and how it is conceived as a drug versus a medication, but it was still difficult to disentangle from street methadone. Prison staff also struggled to separate street and prison methadone. The differences observed evolved from a moral and legal standpoint rather than disentangling how the two substances differed in terms of euphoric effect, how it is taken, and how it is obtained. One participant expressed their frustration that someone could be imprisoned for dealing drugs and then receive methadone legally in prison.

"Well, what do you think if, bloody hell, if the two medics are involved in something like that and then we get this as well. Two people are in for dealing drugs, so here have some methadone as well, like, I haven’t seen anything like that before, I cannot wrap my mind around it… Two staff members at the medical unit are involved in dealing drugs, and they also get a kilo of methadone, here you go, here’s for you to distribute in the framework of some program" —Inessa (prison administration, Prison B).

On the other hand, some prison staff viewed methadone as a purely medical treatment, separate, and different from other illicit drugs in prison.

R: They can’t just get drugs in and inject them without permission. If they want permission, they have to pay.

I: Methadone including? Is methadone included?

R: Methadone is purely… medical… — Leonid (clinical staff, Prison A).

#### Prison life beyond Soviet legacies: shifts away from criminal subculture

Many participants commented on the existence of different prison governing systems. More specifically, the existence of “black” versus “red” prisons, or prisons run primarily by PDL versus prisons run by the formal administration, respectively—a distinction that exists throughout the post-Soviet space. Though the Ukrainian prison system is perceived to have been transitioning more toward “red” prisons that espouse organization and treatment more aligned with Western Europe, a legacy from the Soviet system remains in part in the prison system. Prison staff considered administration-run prisons a more European model and PDL-led prisons a Soviet model of prison governance. Some staff, however, felt that Ukrainian prisons still followed a Soviet model and were, therefore, poorly equipped to host methadone treatment programs:

"You are perfectly aware of what the difference between our Ukrainian and European intellect is. They can give drugs to European convicts and would rather not give them to ours. Because their convicts fear the administration, whereas our convicts can do harm. This is a threat to the lives and health of workers and convicts themselves" —Stefan (clinical staff, Prison A).

Criminal subculture, however, was understood to be shifting toward a “western” model. Although not extinct, the deterioration of the informal prison structure was seen as symptomatic of a shift toward Europe.

“The biggest one [thief-in-law] in the hierarchy is almost gone. We are moving forward, we are going to Europe. And in Europe, there is no such thing” —Vyktor (PDL not enrolled in OAT, 29 years old, Prison A).

Participants often considered this shift away from red and black prisons not only as a movement toward Europe but specifically as a shift away from Ukraine’s Soviet past. This has been an especially salient trajectory for Ukraine since Russia invaded Ukraine in 2014 and illegally annexed Crimea.

“A lot has changed, probably. We became more independent from our Soviet past. From the gulag culture and so on. There is more humanity now” —Stasya (clinical staff, Prison B).

The hierarchy’s perceived diminished influence was understood as a result of this cultural shift.

“I believe that 20 years ago the prison hierarchy was much more rigid, the inmates themselves followed those rules and supported the hierarchy with much more vigor as compared to now. The rules themselves are being simplified, the people are changing, I believe that 20 years ago the inmates much more closely followed the rules and stayed true to the lifestyle. Nowadays, the people have changed, and I know that the hierarchy’s impact has diminished. Perhaps it has changed because the world is changing, and it has impacted the hierarchy” —Nyusha (clinical staff, Prison B).

Some felt that the diminished influence of the hierarchy, due to the observed cultural shift, manifested in the form of more relaxed rules surrounding selling and using drugs among PDL. Additionally, participants felt a more egalitarian social order was forming among the PDL.

"For example, if someone sold drugs, he was considered lower and could no longer consider himself a normal convict. There is no such division now… They used to be called hucksters in prison slang, who had traded in drugs, so they could not talk to others. It was in 2007, 2008, and 2009. Now they are all equal. That’s how it is" —Dobrushin (prison administrator, Prison A).

#### The effects of the Russian invasion on methadone

After the 2022 Russian invasion, many Ukrainian prison populations were displaced and transferred to prisons in more western regions. The local people in prisons (i.e., non-displaced persons) commented on the large swaths of PDL newly placed in their prisons, and they had the perception that there were distinct cultural differences in which the new individuals were more aligned with Russia.

“They [recently transferred people in prison] have a different way of thinking, their mentality is more like Russia. It is the Russian mentality. If Russians had come after them, there was that zone, they would have taken machine guns and gone against Ukrainians. And they were offered methadone, they were given food and water” — Artur (PDL not enrolled in OAT, 25 years old, Prison A).

Other participants describe that these new transfers arrive with withdrawal symptoms, potentially as their illegal drug supplies were interrupted. These individuals are often transferred from prisons that lack methadone treatment programs and are perceived as having a higher need for methadone and, therefore, more readily engaged with the methadone treatment program.

“Well, look, now I'll tell you something interesting. Due to the war, the current one, a zone was transported from Zaporizhzhia. One hundred and twenty people came here… And they all came; Well, not all, but not a small mass with withdrawals. Here they are put on the program, yes. They didn't have this program” —Ehor (PDL enrolled in OAT, 43 years old, Prison B).

New transfers were described as less well-connected than the local PDL. This, in turn, limited new PDL arrivals’ ability to procure drugs illegally. Consequently, their greater participation in the methadone treatment programs was understood to be a result of necessity.

“They [transferred PDLs] came here, again, a new place, and as we said, it's not so easy to get it [methadone]. Especially for the new people, nobody's going to tell them these ways of getting high on methadone, let's say street methadone, nobody's going to tell them. Because new people, everybody's afraid to tell their secrets, right. So it's like, I've seen them feel bad, they've been starting to get kumar, they've realized that they can't find the drug fast enough and so they've decided to get into the programs” —Olek (PDL not enrolled in OAT, 43 years old, Prison A).

New transfers confirmed the perceptions of others as they reported enrolling in the methadone program after their arrival.

“I: So, let me get this straight: you abused [drugs] a lot, you were arrested, you went to jail, where you stopped abusing from one day to the next?

R: No, I stopped using it here in the camp.

I: Oh.

R: Well, I came here from the pre-trial detention center, and I started a program, this one here, methadone.

I: Yeah, yeah.

R: And in the jail, I was using, yes. We've been doing it there, there's no program in the pre-trial facility —Roman (recently transferred PDL enrolled in OAT, 34 years old, Prison B).

## Discussion

Our findings point to new insights into how criminal subculture in Ukrainian prisons relates to illegal drug use and evidence-based treatment. While most participants did acknowledge the existence of an informal hierarchy among PDL, perceptions of its influence and strength of this influence varied and appeared to be waning over time. In some cases, the criminal subculture was understood to vehemently prohibit drug use, particularly among those of a higher caste. In contrast, others felt the criminal subculture was waning and, therefore, had less ability to dictate a person’s within-prison drug use. Whether methadone treatment and even illegal drug use were supported by the informal hierarchy was also ambiguous and in flux, especially as there was the perception of moving toward a more European perspective. This, in part, is reflected in the data in which PDL and prison staff struggled to identify and articulate differences between street and program methadone and how the meaning of street methadone can be transformed when administered in prison settings. Finally, the Russian invasion seemed to impact the uptake of methadone treatment programs as newly transferred PDL were understood to have more need, especially as they were observed to have psychological distress from being on the frontline and experiencing symptoms of withdrawal. Consequently, displaced PDL were more likely to engage with these programs, potentially as the prison subculture did not interfere. It appeared that the informal hierarchy provided some empathy toward them by virtue of their proximity to the war and did not actively dissuade them from treatment. To the best of our knowledge, these were the first interviews ever conducted in Ukrainian prisons during wartime.

While we cannot fully explain why the influence of the criminal subculture is waning, there are a few possible explanations. First, each of the countries of the former Soviet Union has had its own distinct political, economic, and social trajectory, which may, in turn, impact the criminal subculture. For example, the influence of the criminal subculture was nearly erased in Georgia, where there were major economic and anti-corruption activities, which markedly reduced the influence of the prison hierarchies (29). The situation in Ukraine, however, may be different as Ukraine distinguished itself early from Russia through its Orange Revolution in 2004, where it denounced Russia’s influences in its political process. The trajectory of Russia and its continued perpetuation of the criminal subculture departed further when Russia invaded Ukraine in 2014 and annexed Crimea. This departure from Russian policies grew further, as observed here, when Russia invaded Ukraine again in 2022 with a full-scale war.

Parts of our data regarding the criminal subculture in prisons in Ukraine are consistent with the literature from other EECA countries like Moldova and the Kyrgyz Republic, which illustrates how criminal subcultures can inhibit the uptake of prison-based methadone treatment programs (18, 21, 44). Ukraine, however, differs from these countries, where methadone had been introduced in prisons nearly two decades ago. The role of the prison subculture throughout EECA has evolved. At some points, the prison subculture operated the drug trade from an economic perspective. Later, some hierarchies outlawed the trade, but in the case of Kyrgyzstan, the hierarchy provided liquid poppy straw (an opioid) every 10 days as payment for loyal workers.

Findings here, however, resonate with data elsewhere that drug use is prohibited by the thieves’ code and can be actively enforced by those who occupy a higher caste via ostracization (19). As methadone is newly being introduced as a medical treatment, methadone appears to be emerging as a formal version of street methadone, not sanctioned as a medical treatment and therefore something not to be trusted. An alternative explanation for why MMT is mistrusted by some PDL is that the prison subculture often mistrusts any activities they do not control, a finding observed in Kyrgyzstan (45). We found, however, that the thieves’ code was applied more harshly to PDL belonging to higher castes, which limited their participation in methadone treatment programs. Among those that occupied lower castes, there was more flexibility surrounding drug use. There was not a clear consensus on whether using program-delivered methadone was considered against the informal hierarchy’s rules.

Of importance is the observed conflation between “street” and “program” methadone, which may explain the lack of consensus as to whether the program-delivered methadone qualified as an illicit drug or a medication. Even outside prisons, negative attitudes toward methadone as treatment exist (36). Interviewers also struggled to identify which type of methadone (street vs. MMT program) participants were referring to as participants would use the terms interchangeably. Rhodes et al. noted that there is “no single biomedical object of methadone…” in the East African context (46). Our findings echo this sentiment as we illustrate how understandings of methadone traverse various conceptual topographies in Ukrainian prisons, and it can be considered a drug that induces euphoria, an addiction treatment, or a “legal” drug with reduced psychoactive potency. Future prison-based methadone programs should, therefore, consider hosting joint, formal education and counseling sessions with both prison staff and PDL to help distinguish between prison and street methadone and correct any misconceptions about program methadone. Additionally, participants noted that delivering methadone through the prison-based program transformed the experience of the medication itself. Some participants used language that suggests the methadone program is a form of biopower held by the administration that further transforms the object. Specifically, the statement, “he will become vulnerable and weak after joining the treatment program… It’s distributed by the administration. If it was done by an outsourced doctor, well, perhaps it would somehow be different.” In a prison setting, where people with substance use disorders have no choice but to cooperate with those who wield power over them to receive treatment, methadone may be understood as a form of social control over drug users (47, 48). Consequently, the fear of this observed social control both transforms the object of methadone and may dissuade potential participants from engaging in methadone treatment.

The invasion of Ukraine by Russia has the potential to transform both the prison subculture and the perceptions of methadone as a treatment. While the government has clearly aligned itself more with the West during the violent invasion of Ukraine by Russia, it may have the potential to interrupt the existing perception of program methadone being conflated as street methadone and further support its scale-up. At least on the surface, it appears that the early trajectory is to potentially transform methadone as a treatment. However, within prison, training and peer training may be needed as prisons are often insulated from perceptions outside this “hidden” context.

Despite many of our findings aligning with other prison-based methadone program studies, Ukraine’s prison setting was unique as it appeared to be “in transition” away from criminal subculture-dominated prisons and not as easily categorized into black versus red prisons. This may bode well as methadone is scaled up within prisons, and there is the perception that people become healthier. Alternatively, as was observed in Kyrgyzstan, PDL on methadone were also provided “dimedrol” (diphenhydramine), resulting in the deteriorating health of methadone patients and undermining methadone as an effective treatment (49). Previous research on prison structures in Kyrgyzstan notes that national political stability and regimen transitions are often mirrored within the prison walls of a nation (50). Our qualitative data reveal a similar pattern within Ukrainian prisons, and methadone treatment may, therefore, become intertwined with local politics. Not only did participants observe and report shifts away from informal prison hierarchies, but they also explicitly mapped this diminishing structure as Ukraine left its Soviet past behind and moved toward Europe. Outside of prison facilities, Ukraine’s shift toward Europe has also materialized in its pursuit of European Union (EU) and NATO memberships (51, 52). This finding underscores the importance of understanding inter- and intra-national politics that may impact the implementation of health interventions.

Finally, we explored the effects of the Russian invasion on the Ukrainian prison-based methadone treatment programs. We found that recently transferred PDL were perceived as more aligned with Russian identity and more likely to participate in these programs. Non-displaced PDL understood this to be a result of necessity, as newly transferred PDL were less likely to have existing social ties that would enable them to access drugs through other means. This finding may also be reflective of Emergent Norm Theory, which stipulates that “non-traditional behavior develops in crowds as a result of the emergence of new behavioral norms in response to a precipitating crisis (53).” While our data, coupled with Emergent Norm Theory, suggest methadone treatment is frowned upon by some PDL in this setting, newly transferred individuals have not yet been socialized in this environment. They are, therefore, unimpacted by predominant norms, which may explain relatively high perceived uptake.

While prison transfers across borders heightened perceived participation in methadone programs, the Russian invasion of Ukraine placed the country’s methadone programs in peril (54). Within a week of Russia’s illegal annexation of Crimea in 2014, all methadone clinics in the region were permanently closed. The closures left 800 patients stabilized on methadone at the time without access to life-saving medication. Many of the methadone patients chose to migrate to non-occupied territories. For 10% of the patients, however, the closures proved fatal as withdrawal from methadone led to suicide, opioid relapse, and fatal overdose (55). While access to methadone has temporarily increased for those transferred out of Ukraine, within their own country, methadone is in danger of prohibition, a devastating outcome that will increase rates of HIV/HCV, overdose, and unregulated injection drug use and permanently deny access to life-saving medication.

Despite these important findings, there are some limitations. First, we only conducted interviews with PDL and staff from two prisons in the western region. These two prisons were the first ones to introduce methadone after the initial pilot program that has since closed. Qualitative data are highly contextual and hypothesis-generating, so our findings may not be generalizable to other PDL. Second, social desirability bias may have impacted the data gathered. Despite interviewers making it clear that they were not associated with the prison administration, PDL may have assumed that research staff would share information about drug use with the formal administration. Consequently, participants may have been less likely to disclose details of their lives and habits that went against the law.

## Conclusion

The evolving relationship between criminal subculture and a nascent methadone program in Ukrainian prisons appears to impact the understanding and uptake of MMT programs among PDL. During this process, it will be important to initiate education programs for PDL and custodial staff to recognize that two forms of methadone exist and compare and contrast them in terms of their impact on health, with one having greater potential to harm if not dosed adequately. The Russian invasion also provides a unique context for program rollout as it may function to rapidly shape methadone into a more well-defined object. It is critical that as prison-based programs continue to grow, they account for the changing influences of criminal subculture as Ukraine progressively makes political and social shifts toward the West.

## Data availability statement

The raw data supporting the conclusions of this article will be made available by the authors, without undue reservation.

## Ethics statement

The studies involving humans were approved by Ukrainian Institute on Public Health Policy IRB and Yale University IRB. A certificate of confidentiality was provided by the National Institutes of Health to protect participants. The studies were conducted in accordance with the local legislation and institutional requirements. The participants provided their written informed consent to participate in this study. Written informed consent was obtained from the individual(s) for the publication of any potentially identifiable images or data included in this article.

## Author contributions

FLA and LA contributed to the conception and design of the study as they have conducted research on the criminal subculture in Moldova and the Kyrgyz Republic previously. IP, TK, and NS adapted the protocol and arranged the fieldwork. LA, MT, and DB conducted the interviews. MP, LA, MT, and DB performed the qualitative analysis. MP wrote the first draft of the manuscript. All authors contributed to the article and approved the submitted version.
