# Supplementary material for: Introducing methadone maintenance therapy into Ukrainian prisons: a qualitative study of criminal subculture, Russia’s full-scale invasion, and contested methadone objects
Source: Front Psychiatry. 2023 Nov 30;14:1227216. doi: 10.3389/fpsyt.2023.1227216 (PMC10720714; doi:10.3389/fpsyt.2023.1227216)
Supplement: Supplementary file 1 [file Table_1.DOC]

- **WE ARE INTERESTED IN HOW ACCOUNTS MAKE/DEPICT**: Methadone; Illegal drugs; Methadone treatment; Criminal subculture; the Ukrainian prison setting; prisoners, other key actors in the prison (including researchers, medical and administrative staff); Evidence about these; addiction and health and its improvement.
- **WHAT are the OBJECTS and SUBJECTS being said?** [What is the ‘evidence’, ‘methadone’, ‘drugs’, ‘prisoner,’ ‘criminal code’ etc which is being represented?]
- **HOW are these OBJECTS and SUBJECTS made possible?** [What resources, discourses, evidence, actors, etc, are used to substantiate what is being represented?]
- **HOW does the account PROBLEMATISE the issue at hand?** [What is the ‘problem’ represented to be, and its ‘solution’?]
- **What is made ABSENT or SILENCED by the account?** [What is missing, and what is pushed into the background?]

| **Background/Role** | **Ukrainian prisons** | **Methadone** | **Drug use** | **Criminal Subculture** | **Future** |
| --- | --- | --- | --- | --- | --- |
| Daily life incl. before prison  Involvement in MT and drug use  How came to be involved  Health and healthcare  History of drug treatment  Expectations for life post-release | Tell me about Ukrainian prisons  Situation in relation to HIV, drugs, prisons, and MT  What addiction programmes currently work well and do not work well  What programmes are needed  How are these evidenced  Relationships with on-the-ground NGOs, prison leadership (both formal and informal), prisoners  Experience navigating between the formal and informal in daily interactions | How do you see MT  What is it, and how does it work, how effective  What do we know about its effects? And how?  What is the role of MT (prison, beyond)  What is the need for MT in Ukrainian and Ukrainian prisons  What are the challenges in implementing MT  Other actors involved in MT  Particular examples of interaction with MT | Tell me about drug use in and outside of prison  How does it work  What motivates it  What do we know about effects?  How do we know this?  What should be done about it?  Other actors involved in drug use  Particular examples of interactions with drug use | Tell me about hierarchical divisions  What do they tell us  How do they work?  What are their implications?  How do they relate to MT?  Other drugs?  What do they tell us about the challenges of implementing MT and other addiction treatment in prison settings | Tell me what needs to happen going forwards:   - Methadone - Drug use - Criminal subculture   What has been unexpected for you?  What perspectives of yours have changed?  How can interventions (MT, or others) respond to its implementation challenges?  How can/should policy change?  What should the role of methadone/prison formal and informal leadership be? |

- **What are the CONTRADICTIONS in, and ALTERNATIVES to, what is being represented?** [Make visible the tensions/contradictions in different ways of seeing things]
- **What are the IMPLICATIONS?** [What are the implications of representing things in particular ways, for subjects, for health, for intervention]
